# The Repurposing of Anti-Psychotic Drugs, Quetiapine and Olanzapine, as Anti-Cryptococcus Drugs

**DOI:** 10.3389/fmicb.2017.00815

**Published:** 2017-05-09

**Authors:** Adepemi O. Ogundeji, Carolina H. Pohl, Olihile M. Sebolai

**Affiliations:** Department of Microbial, Biochemical and Food Biotechnology, University of the Free StateBloemfontein, South Africa

**Keywords:** cryptococcus, macrophage, olanzapine, repurposing, quetiapine

## Abstract

The management of cryptococcal infections is often difficult. This can, in part, be attributed to the fungistatic nature of fluconazole, which may result in cells disseminating to give rise to pathogen-emergent psychosis following brain inflammation. This chance at treatment failure has necessitated the current study wherein the antimicrobial quality of anti-psychotic drugs viz. quetiapine and olanzapine, was assessed. The response of test strains toward quetiapine or olanzapine alone and in combined therapy with fluconazole or amphotericn B was measured. In addition, the mode of action of the two anti-psychotic drugs in killing cryptococcal cells was determined. At the end, the ability of these anti-psychotic drugs to chemo-sensitize macrophages was also examined. The assessed strains were shown to be susceptible to the two anti-psychotic drugs, which possibly killed them via altering their membrane function. Additionally, these anti-psychotic drugs acted in synergy with fluconazole and amphotericin B in controlling the growth of the test strains. Importantly, these drugs improved the phagocytic efficiency of macrophages and, at the same time, stimulated them to produce pro-inflammatory cytokines (interleukin 6 and interferon gamma), said to be critical in the clearance of cryptococcal cells. The minimum inhibition concentration of each anti-psychotic drugs was calculated to be within its respective recommended therapeutic range. This study's findings highlight the potential clinical application of quetiapine and olanzapine as alternative anti-*Cryptococcus* drugs, which can be used to manage the fungal burden (infection) as well as the associated symptom (psychosis).

## Introduction

*Cryptococcus (C.) neoformans* is an important fungal pathogen that causes cryptococcal meningoencephalitis (Park et al., 2009). This fatal inflammatory condition typically manifests in HIV-infected persons with a CD4^+^ T-cell count that is <100 cells/μL (Bicanic and Harrison, 2004). For the condition to arise, cryptococcal cells should disseminate from the lungs and cross the blood-brain barrier (Casadevall, 2010). To be specific, cells are reported to invade macrophages and, in a Trojan horse-like manner, use these immune cells to cross the blood-brain-barrier (Casadevall, 2010). In the brain, the cells compromise the ability of the brain to reabsorb the cerebrospinal fluid, leading to internal accumulation (Adams, 2016). The resultant intracranial pressure may leave patients with adverse neurological signs such as psychosis (Bicanic and Harrison, 2004). Without treatment such a patient is expected to die within 3 months (Perfect et al., 2010).

Although guidelines for the management of cryptococcal diseases have been published (Perfect et al., 2010), management still remains difficult, more so in resource-poor countries, due to cost. In addition, the rise in drug resistance, and inability of the current anti-fungals to discriminate pathogen targets from the host's (both are of eukaryotic origin)—implies that these drugs will often lead to clinical failure (Ghannoum and Rice, 1999; Armstrong-James et al., 2014). This inability to discriminate has unfortunately also led to stagnation in the development of new anti-fungals. To illustrate this point, it is not surprising that the last anti-fungal drug to be placed on the market was ~20 years ago (California Biomedical Research Association, 2012).

A possible solution to overcome some of these shortcomings may be to repurpose already FDA-approved drugs that are typically prescribed to treat non-infectious conditions. Therefore, we considered repurposing two anti-psychotic drugs *viz*. quetiapine and olanzapine, as candidate anti-*Cryptococcus* drugs. The findings of this study could potentially be of clinical use in two ways: (1) kill disseminated cryptococcal cells, and (2) manage pathogen-emergent psychosis.

## Materials and methods

### Cultivation and standardization of cells

#### Fungi

Five *C. neoformans* clinical strains (LMPE 028, LMPE 030, LMPE 043, LMPE 046 and LMPE 047) and five *Cryptococcus gattii* clinical strains (LMPE 045, LMPE 048, LMPE 052, LMPE 054, and LMPE 070), which were obtained from Universitas Academic Hospital (South Africa), were used in the study. These strains were grown on yeast-malt-extract (YM) agar (3 g/l yeast extract, 3 g/l malt extract, 5 g/l peptone, 10 g/l glucose, and 16 g/l agar; Merck, South Africa) at 30°C for 48 h. Five colonies (from each respective agar plate) were scraped off and suspended in 10 ml of distilled water or RPMI-1640 medium (Sigma-Aldrich, South Africa). At the end, the cells were standardized to prepare final inocula of between 0.5 × 10^5^ and 2.5 × 10^5^ CFU/ml according to European Committee on Antimicrobial Susceptibility Testing (EUCAST) guidelines (Arendrup et al., 2015). The inocula were kept on ice before use.

#### Immune cells

The macrophage cell line RAW 264.7 (a kind donation from Prof. Masoko and Mr. Makola, University of Limpopo, South Africa), was used in the study. Cells were grown using RPMI-1640 medium that was supplemented with 10% fetal bovine serum (Biochrom, Germany), 20 U/ml penicillin (Sigma-Aldrich, USA), 20 g/ml streptomycin (Sigma-Aldrich, USA), and 2 mM L-glutamine (Sigma-Aldrich, South Africa) in a 5% CO_2_-incubator (Thermo Fisher Scientific, USA) at 37°C until they reached 80% confluence. Thereafter, the cells were standardized to 1 × 10^5^ cells/ml and seeded into wells of a sterile, disposable 96-well flat-bottom microtiter plate (Greiner Bio-One, Germany).

### Drugs

Standard powders of quetiapine (Sigma-Aldrich, South Africa), olanzapine (Sigma-Aldrich, South Africa), fluconazole (Sigma-Aldrich, South Africa) and amphotericin B (Sigma-Aldrich, South Africa) were used in this study. Quetiapine and olanzapine were prepared in dimethyl sulfoxide (Merck, South Africa) to each yield a stock solution of 1,000 mg/ml. Fluconazole was reconstituted in distilled water (final stock solution of 1,000 mg/ml) while amphotericin B was dissolved in dimethyl sulfoxide (DMSO) (Merck, South Africa) to yield a stock concentration of 1,000 mg/ml. The concentrations of drug diluents, in which the stock solutions were prepared, never exceeded 1%.

### Drug susceptibility testing

The testing was performed in sterile, disposable 96-well flat-bottom microtiter plates and according to EUCAST guidelines. In short, 100 μl of the standardized inoculum (between 0.5 × 10^5^ and 2.5 × 10^5^ CFU/ml) was aliquoted to designated wells. The cells were then treated with 100 μl of the test drug(s) at twice its desired final concentration. The following final drug concentrations were used, for quetiapine: 0.0625, 0.125, 0.25, 0.5, and 1 mg/ml and for olanzapine: 0.003125, 0.00625, 0.0125, 0.025, and 0.05 mg/ml. The plate was incubated for 48 h at 37°C. After 48 h, the optical density (OD) of each well was measured at 562 nm using a spectrophotometer (Biochrom EZ Read 800 Research, United Kingdom). In the study, the MIC was defined as the lowest drug concentration that resulted in 50% or more growth inhibition compared to drug-free control.

All subsequent tests, which are detailed below, were carried out on the one fungal strain that showed the greatest sensitivity toward all test drugs. In addition, fluconazole and amphotericin B MICs were based on those reported by Ogundeji et al. (2016). The Ogundeji et al. study and the current study were done at the same time, in the same laboratory. Thus, in this study, the MIC for fluconazole was considered as 8 mg/ml while for amphotericin B it was 1 mg/ml.

Four checkerboard assays were prepared i.e., quetiapine-fluconazole, quetiapine-amphotericin B, olanzapine-fluconazole, and olanzapine-amphotericin B in sterile, disposable 96-well flat-bottom microtiter plates. These drug combinations were used to treat the standardized inoculum. The plates were incubated for 48 h at 37°C. At the end of the incubation period, OD readings were taken, and subsequently the fractional inhibitory concentration (FIC) index (FICI) was calculated. Fractional inhibitory concentration index (that is, the sum of the FICs [ΣFIC]) was defined as FIC_A_ + FIC_B_, where FIC_A_ is the MIC of drug A in combination/MIC of drug A alone and FIC_B_ is the MIC of drug B in combination/MIC of drug B alone (Ogundeji et al., 2016). Fractional inhibitory concentration index values were determined to establish if there was synergism (≤0.5), no interaction (>0.5–4) or antagonism (>4).

### Effect of quetiapine and olanzapine on cellular ultrastructure

Cells for scanning electron microscopy (SEM) were obtained from 48-h old non-treated cells (0 mg/ml), quetiapine-treated cells (at determined MIC of 0.5 mg/ml) and olanzapine-treated cells (at determined MIC of 0.025 mg/ml). These cells were prepared as detailed for drug sensitivity testing assay. They were aspirated and separately transferred to 1.5 ml Eppendorf tubes (Merck, South Africa). The cells were then prepared for SEM according to van Wyk and Wingfield (1991). In short, the cells were chemically fixed using sodium-phosphate-buffered 3% glutaraldehyde (Merck) and sodium-phosphate-buffered 3% osmium tetroxide (Merck, South Africa) followed by dehydration in a graded ethanol (Merck, South Africa) series. Following that, the cells were dried (Bio-Rad Microscience Division, England), mounted on stubs, and coated with gold using an SEM coating system (Bio-Rad Microscience Division, England; van Wyk and Wingfield, 1991). Preparations were examined using a Shimadzu Superscan SSX 550 scanning electron microscope (Japan). In addition, the diameters of 100 cells per each experimental condition (randomly selected from different locations acquired from different stubs) were measured using a ruler application that is coupled to the microscope.

### Effect of quetiapine and olanzapine on membrane function

Cells were prepared as detailed for drug sensitivity assay. Non-treated cells were included as control. For Toxilight® bioassay, the supernatant (20 μl) was collected from the wells and separately transferred to wells of a sterile, white 96-well flat-bottom microtiter plate (Greiner Bio-One, Germany). Next, 100 μl of the Toxilight reagent (Lonza, USA) was added to all the wells. The plate was incubated for 5 min at 37°C. To quantify the amount of adenylate kinase released from cells with damaged membranes, the bioluminescence generated from each well was measured using a Fluoroskan Ascent FL (Thermo-Scientific, USA) microplate reader, which converted logarithmic signals to relative luminescence units.

For propidium iodide (PI) staining assay, the plate was briefly agitated in order to re-suspend the cells in the cultivation media. Next, 99 μl of the re-suspended cells (from indicated wells) were separately transferred to a sterile, black 96-well flat-bottom microtiter plate (Greiner Bio-One, Germany). Following this, 1 μl of the PI (Life Technologies, USA) stain was added to designated wells to initiate the reaction with cells. The plate was immediately incubated in the dark for 30 min at 37°C. To measure the amount of PI that permeated through damaged membranes, the stain was excited at 485 nm and the corresponding emitted fluorescence signal was read at 538 nm using the Fluoroskan Ascent FL microplate reader.

### Effect of quetiapine and olanzapine on macrophages

#### Effect on macrophage growth

Standardized macrophages were first seeded and grown overnight in a sterile, disposable 96-well flat-bottom microtiter plate. The next day, the media was aspirated and fresh media (100 μl) was added to the wells. The cells were then challenged with either 100 μl of twice the desired final concentration of quetiapine (1 mg/ml) or olanzapine (0.05 mg/ml). The plate was then incubated for 48 h at 37°C in a 5% CO_2_ incubator. The OD of the cells was measured at 562 nm using a Biochrom spectrophotometer. The non-treated cells were included as control.

To complement the OD readings, the metabolic activity of these cells was also measured. In short, a duplicate plate was prepared as stated above. After a 48 h incubation period, the cells were reacted with 54 μl of 2,3-bis (2-methoxy-4-nitro-5-sulfophenyl)-5-[(phenylamino)carbonyl]-2H-tetrazolium hydroxide (XTT; Sigma-Aldrich, South Africa) in the presence of 1 mM of menadione (Sigma-Aldrich, South Africa). The plate was accordingly incubated in the dark in a 5% CO_2_ incubator. Three hours after initiating the tetrozolium reaction, the OD of the wells was finally measured at 492 nm using a Biochrom spectrophotometer. The non-treated cells were included as control.

#### Effect on immunological function

To characterize the immunological response of macrophages to cryptococcal cells, in the presence of test drugs, ELISA assays were performed. In short, standardized, seeded macrophages were incubated overnight in a 5% CO_2_ incubator. The next day, the media was aspirated and fresh media (100 μl) was added to the wells. Standardized cryptococcal cells in 100 μl of RPMI 1,640 medium containing twice the desired final concentration of either quetiapine (1 mg/ml) or olanzapine (0.05 mg/ml) were added to macrophage wells to prepare co-cultures (1:1 effector-to-target ratio). Cells were allowed to interact for 6 h before ELISA assays were performed. Co-cultures prepared in the absence of test drugs were included as controls. The supernatant was then aspirated and used for interferon-gamma (IFN-γ; BioLegend, USA) and interleukin-6 (IL-6; BioLegend, USA) ELISA assays. Each ELISA assay was performed according to its manufacturer's instructions. At the end, all plates were read at 450 nm on the Biochrom spectrophotometer. Where applicable, concentrations were extrapolated from the constructed standard curve.

#### Effect on macrophage phagocytic function

The ability of macrophages to internalize cryptococcal cells was measured using the phagocytosis stain, pHrodo^TM^ Green Zymosan A BioParticles (Life Technologies, USA). This stain only fluoresces when excited at acidic pH, such as inside the lumen of phagolysosomes. Standardized cryptococcal cells in 999 μl of RPMI 1640 medium, were reacted with 1 μl of the stain in 1.5 ml Eppendorf tubes for 1 h at 37°C while slowly agitating. Next, the cryptococcal cells were washed twice with PBS, spun down and suspended in 1,000 μl of fresh media that contained twice the desired final concentration of either quetiapine (1 mg/ml) or olanzapine (0.05 mg/ml). A 100 μl suspension of cells was then immediately aliquoted to wells that contained seeded macrophages (100 μl) to prepare a co-culture (1:1 effector-to-target ratio) in a microtiter plate. The plate was incubated for 6 h at 37°C in a 5% CO_2_ incubator. At the end of the incubation period, the induced fluorescence was measured (492 nm; ex/538 nm; em) using a Fluoroskan Ascent FL microplate reader. Fluorescence was also measured for non-treated co-cultures i.e., co-cultures without drugs.

In addition, the ability of cytokine standards i.e., IFN-γ (500 pg/ml) and IL-6 (500 pg/ml) to activate macrophages to internalize cryptococcal cells was also measured. Here, cytokines substituted the test drugs stated above. Likewise, the cryptococcal cells were stained and the co-culture prepared similarly. The plate was also handled in the same manner before taking measurements on the plate reader. Fluorescence was also measured for co-cultures without cytokines.

### Statistical analysis

All data, unless stated otherwise, represent mean values of three biological replicates. To show the statistical significance of data between the different experimental conditions, the standard deviations and Student *t*-tests were determined using Microsoft Excel. A *p*-value equal or below 0.05 was regarded as statistically significant.

## Results

### Quetiapine and olanzapine possess anti-fungal activity and act in synergy

All ten *Cryptococcus* strains (five *C. neoformans* and five *C. gattii*) showed a dose-dependent growth reduction pattern toward the two test anti-psychotic drugs when compared to their respective drug-free controls (Tables 1, 2). The MIC of quetiapine was defined as 0.5 mg/ml while that of olanzapine was 0.025 mg/ml. At these respective concentrations, both drugs effected a 50% or more growth reduction when compared to their respective drug-free controls. More importantly, each defined MIC was within the recommended therapeutic range in the blood, for each respective drug (Skov et al., 2015; Lu et al., 2016). Outside the therapeutic dosage, greater growth reduction could be achieved. However, this is not ideal as this will result in ill-tolerance by patients. The strain *C. neoformans* LMPE 046 was the most sensitive toward all test drugs, including fluconazole and amphotericin B (Ogundeji et al., 2016). Thus, all subsequent results were based on the response of this one fungal strain at the above-mentioned MICs.

**Table 1:** **The effect of quetiapine on ***C. neoformans*** and ***C. gattii*** strains**.

**Species detail**		**Drug response**					
		**Non-treated cells**	**Quetiapine (mg/ml)**				
			**0.0625**	**0.125**	**0.25**	**0.5 (MIC)**	**1**
**Name**	**Number**	**OD**_562nm_	**%GR**	**%GR**	**%GR**	**%GR**	**%GR**
*C. neoformans*	LMPE 028	0.535 (0.021)	24 (0.005)	33 (0.011)	44 (0.011)	54 (0.006)	76 (0.008)
*C. neoformans*	LMPE 030	0.536 (0.005)	22 (0.012)	34 (0.050)	45 (0.017)	55 (0.012)	75 (0.012)
*C. neoformans*	LMPE 043	0.534 (0.016)	23 (0.040)	33 (0.015)	44 (0.015)	55 (0.008)	75 (0.005)
*C. neoformans*	LMPE 046	0.534 (0.008)	26 (0.083)	35 (0.012)	47 (0.009)	57 (0.032)	79 (0.014)
*C. neoformans*	LMPE 047	0.533 (0.013)	23 (0.049)	34 (0.051)	45 (0.013)	56 (0.016)	74 (0.009)
*C. gattii*	LMPE 045	0.532 (0.016)	22 (0.016)	30 (0.015)	43 (0.008)	53 (0.024)	72 (0.023)
*C. gattii*	LMPE 048	0.530 (0.040)	25 (0.008)	33 (0.021)	45 (0.014)	56 (0.009)	76 (0.006)
*C. gattii*	LMPE 052	0.530 (0.008)	22 (0.013)	33 (0.014)	43 (0.009)	54 (0.014)	73 (0.005)
*C. gattii*	LMPE 054	0.531 (0.005)	22 (0.008)	31 (0.008)	42 (0.015)	54 (0.011)	74 (0.006)
*C. gattii*	LMPE 070	0.534 (0.017)	21 (0.019)	30 (0.019)	43 (0.012)	53 (0.012)	72 (0.017)

*Percent growth reduction was calculated as 100%—[(OD of treated cells/OD of non-treated cells) 100%]. Values represent the mean values from three biological replicates, and values in parentheses represent standard deviations. %GR = Percentage growth reduction*.

**Table 2:** **The effect of olanzapine on ***C. neoformans*** and ***C. gattii*** strains**.

**Species detail**		**Drug response**					
		**Non-treated cells**	**Olanzapine (mg/ml)**				
			**0.003125**	**0.00625**	**0.0125**	**0.025 (MIC)**	**0.05**
**Name**	**Number**	**OD**_562nm_	**%GR**	**%GR**	**%GR**	**%GR**	**%GR**
*C. neoformans*	LMPE 028	0.533 (0.009)	15 (0.015)	28 (0.013)	43 (0.021)	56 (0.014)	63 (0.014)
*C. neoformans*	LMPE 030	0.531 (0.014)	16 (0.014)	27 (0.040)	41 (0.007)	54 (0.007)	62 (0.009)
*C. neoformans*	LMPE 043	0.538 (0.005)	18 (0.008)	28 (0.005)	42 (0.005)	55 (0.018)	64 (0.012)
*C. neoformans*	LMPE 046	0.532 (0.032)	19 (0.042)	30 (0.022)	44 (0.009)	58 (0.009)	67 (0.007)
*C. neoformans*	LMPE 047	0.531 (0.012)	18 (0.009)	29 (0.041)	43 (0.012)	56 (0.018)	65 (0.014)
*C. gattii*	LMPE 045	0.531 (0.026)	13 (0.018)	24 (0.017)	40 (0.013)	52 (0.014)	64 (0.013)
*C. gattii*	LMPE 048	0.530 (0.036)	15 (0.009)	26 (0.023)	42 (0.008)	55 (0.009)	66 (0.018)
*C. gattii*	LMPE 052	0.530 (0.009)	14 (0.011)	25 (0.015)	41 (0.020)	54 (0.013)	63 (0.014)
*C. gattii*	LMPE 054	0.531 (0.015)	13 (0.009)	24 (0.013)	40 (0.011)	52 (0.014)	63 (0.024)
*C. gattii*	LMPE 070	0.534 (0.007)	14 (0.021)	25 (0.009)	40 (0.009)	54 (0.009)	64 (0.007)

*Percent growth reduction was calculated as 100%—[(OD of treated cells/OD of non-treated cells) 100%]. Values represent the mean values from three biological replicates, and values in parentheses represent standard deviations. %GR = Percentage growth reduction*.

When paired with either fluconazole or amphotericin B, each anti-psychotic drug could effect a synergistic outcome (Tables 3, 4). However, no drug combination, within the FIC index, yielded total reduction of fungal growth. Nonetheless, a two-fold downward shift in the concentration (of each test drug—including fluconazole or amphotericin B), that effected a 50% or more growth reduction was observed. The latter is critical as it implies a lower dosage may be required to yield the same desired outcome—this could assist in minimizing adverse effects that are expressed at higher concentrations.

**Table 3:** **Combined effects of quetiapine and Amphotericin B and quetiapine and fluconazole on ***C. neoformans*** strain LMPE 046**.

**Quetiapine (mg/ml)**	**Percentage (%) growth reduction**					**Fractional inhibitory concentration (FIC) index**				
	**Amphotericin B (mg/ml)**					**Amphotericin B (mg/ml)**				
	**0.25**	**0.5**	**1 (MIC)**	**2**	**4**	**0.25**	**0.5**	**1 (MIC)**	**2**	**4**
**0.0625**	23	41	59	68	80	2.21	0.61	0.41	0.35	0.28
**0.125**	35	48	62	74	88	1.91	0.59	0.4	0.3	0.22
**0.25**	48	56	70	79	92	0.63	0.44	0.32	0.28	0.16
**0.5 (MIC)**	61	68	79	83	95	0.4	0.24	0.31	0.23	0.13
**1**	79	81	85	91	98	0.39	0.2	0.24	0.17	0.07
**Quetiapine (mg/ml)**	**Percentage (%) Growth reduction**					**Fractional inhibitory concentration (FIC) index**				
	**Fluconazole (mg/ml)**					**Fluconazole (mg/ml)**				
	**2**	**4**	**8 (MIC)**	**16**	**32**	**2**	**4**	**8 (MIC)**	**16**	**32**
**0.0625**	21	39	54	65	75	2.23	0.62	0.42	0.37	0.3
**0.125**	30	45	59	71	79	2.01	0.55	0.4	0.32	0.25
**0.25**	46	51	68	77	83	0.86	0.44	0.38	0.34	0.21
**0.5 (MIC)**	59	64	74	80	89	0.48	0.38	0.31	0.25	0.19
**1**	72	79	81	89	92	0.31	0.28	0.21	0.19	0.1

*The fluconazole and amphotericin B MICs were based on Ogundeji et al. (2016) study. Shading indicates synergism. The other shaded cells represent the corresponding % growth reduction values in relation to the FICI*.

**Table 4:** **Combined effects of olanzapine and amphotericin B and olanzapine and fluconazole on ***C. neoformans*** strain LMPE 046**.

**Olanzapine (mg/ml)**	**Percentage (%) growth reduction**					**Fractional inhibitory concentration (FIC) index**				
	**Amphotericin B (mg/ml)**					**Amphotericin B (mg/ml)**				
	**0.25**	**0.5**	**1 (MIC)**	**2**	**4**	**0.25**	**0.5**	**1 (MIC)**	**2**	**4**
**0.003125**	19	35	59	69	79	2.69	0.82	0.42	0.33	0.22
**0.00625**	29	43	62	73	86	2.24	0.65	0.39	0.28	0.18
**0.0125**	45	56	68	78	90	0.71	0.43	0.33	0.23	0.16
**0.025 (MIC)**	58	64	75	83	93	0.44	0.35	0.25	0.2	0.12
**0.05**	69	77	80	88	96	0.32	0.29	0.23	0.18	0.09
**Olanzapine (mg/ml)**	**Percentage (%) growth reduction**					**Fractional inhibitory concentration (FIC) index**				
	**Fluconazole (mg/ml)**					**Fluconazole (mg/ml)**				
	**2**	**4**	**8 (MIC)**	**16**	**32**	**2**	**4**	**8 (MIC)**	**16**	**32**
**0.003125**	16	32	53	67	75	2.89	0.89	0.41	0.35	0.24
**0.00625**	27	40	57	70	80	2.41	0.71	0.39	0.32	0.21
**0.0125**	42	52	64	75	86	0.75	0.44	0.32	0.25	0.19
**0.025 (MIC)**	57	61	72	80	90	0.46	0.38	0.29	0.22	0.14
**0.05**	67	75	78	85	93	0.37	0.3	0.25	0.2	0.11

*The fluconazole and amphotericin B MICs were based on Ogundeji et al. (2016) study. Shading indicates synergism. The other shaded cells represent the corresponding % growth reduction values in relation to the FICI*.

### Quetiapine and olanzapine treatment compromises fungal cell wall function

**Figure 1:**
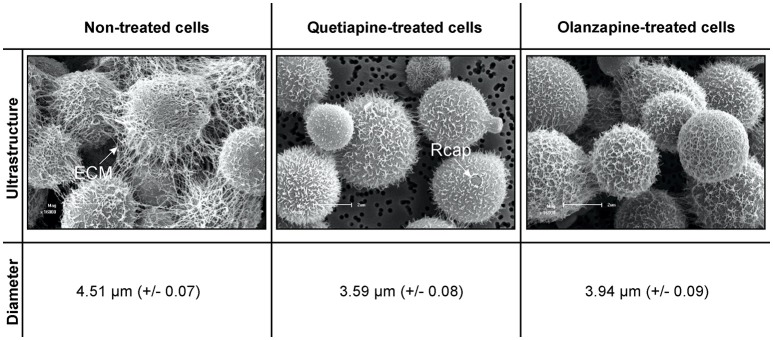
**The effects of quetiapine and olanzapine on the ultrastructure of treated cryptococcal cells as well as on cell size**. For comparison, a SEM micrograph of non-treated cryptococcal cells and their measured cell sizes are included. ECM, extracellular matrix; Rcap, raptured capsule.

A close examination of SEM images revealed the topography of quetiapine-treated cells and olanzapine-treated cells to be different in appearance when compared to that of non-treated cells (Figure 1). Non-treated cells have more web-like extracellular matrixes on their cell wall surfaces when compared to treated cells, which had less web-like structures. The observed reduction in matrixes, may compromise the defenses of cryptococcal cells and may leave them vulnerable to macrophage action. In addition, the treated cells were significantly smaller (*p* < 0.05) in cell diameter (quetiapine-treated cells = 3.59 μm ± 0.08; olanzapine-treated cells = 3.94 μm ± 0.07) when compared to non-treated cells (4.51 μm ± 0.07) (Figure 1).

**Figure 2:**
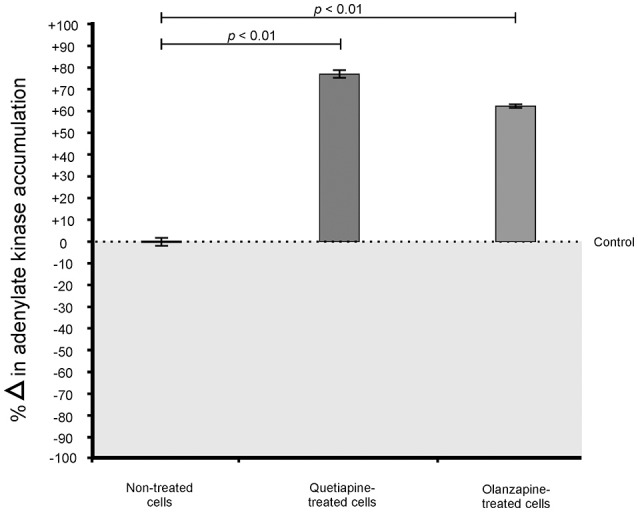
**Toxilight® Bioassay results of treated- and non-treated cryptococcal cells**. When exposed to quetiapine and olanzapine, cells significantly (*p* < 0.01) secreted more intracellular metabolites (adenylate kinase) compared to non-treated cells.

**Figure 3:**
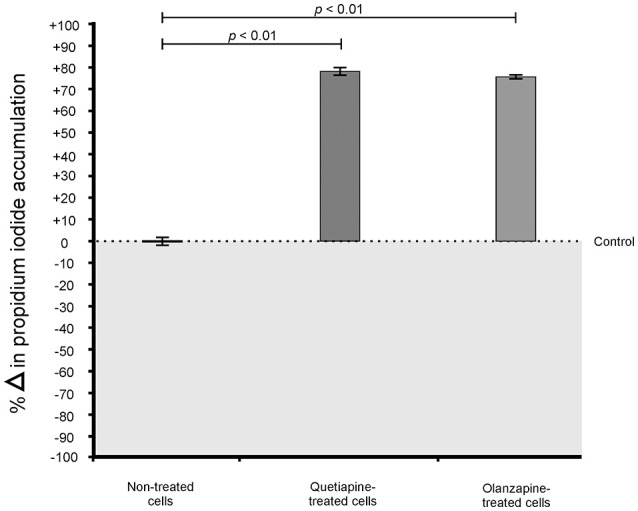
**Propidium iodide (PI) assay results of treated- and non-treated cryptococcal cells**. When exposed to quetiapine and olanzapine, cells significantly (*p* < 0.01) accumulated more PI compared to non-treated cells.

When considering the permeability assay results, it was evident that drug-treated cells significantly (*p* < 0.05) leaked intracellular metabolites, specifically adenylate kinase, into the culture media (Figure 2) and accumulated the PI stain in the cytoplasm (Figure 3), compared to non-treated cells. Both these results (Figures 2, 3) speak to cell walls losing their ability to control the trafficking of molecules in and out of the cells. It reasonable to conclude that the rapture sites on the cell walls, as seen from the topography of quetiapine-treated cells in Figure 1, may be the site where molecules are leaked.

### Quetiapine and olanzapine treatment improves macrophage function

While it was important to demonstrate *in vitro* susceptibility, it was equally important in this study to test the effect(s) of the anti-psychotic drugs on macrophages, which are central for “driving” disseminated infections. Thus, the two test anti-psychotic drugs were chosen particularly because of their lipophilic nature (Bartos and Knudsen, 2013; Natarajan et al., 2016), a quality that may assist these drugs to cross into the lumen of macrophages—thus influence the functioning of macrophages.

**Figure 4:**
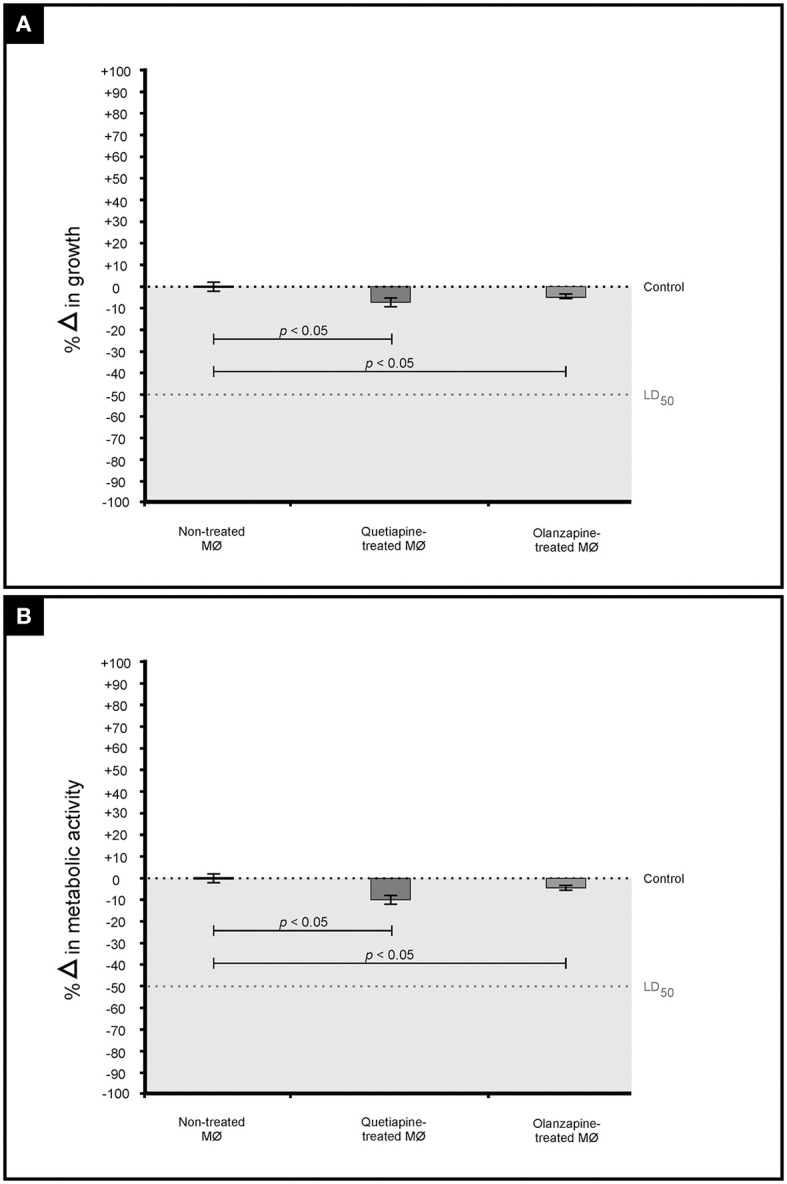
**The graph illustrates the effect of quetiapine and olanzapine on macrophage (MØ) growth (expressed as percentage change in growth) (A)** as well as their effect on metabolic activity (expressed as percentage change in metabolic activity) **(B)**. When considering the results shown in **(A,B)**, it is evident that the two test drugs did not negatively affect macrophages.

It was first sought to determine if the two drugs, at their determined MICs, may negatively affect the growth and metabolic activity of macrophages (Figure 4). When compared to non-treated macrophages, the drug-treated macrophages showed a reduction in both growth (Figure 4A) and metabolic activity (Figure 4B). However, the observed reduction (both growth and metabolic activity) was not significant to reach levels required to effect a lethal dosage (LD_50_)—wherein 50% of the macrophages would be adversely affected. This finding (based on three biological replicates) implies that a mammalian host would not experience negative effects when exposed to the test drugs. This is in line with the determination that the defined MICs, for both drugs, were within the recommended range. Nonetheless, more biological replicates ought to be considered before such a concrete conclusion is drawn.

**Figure 5:**
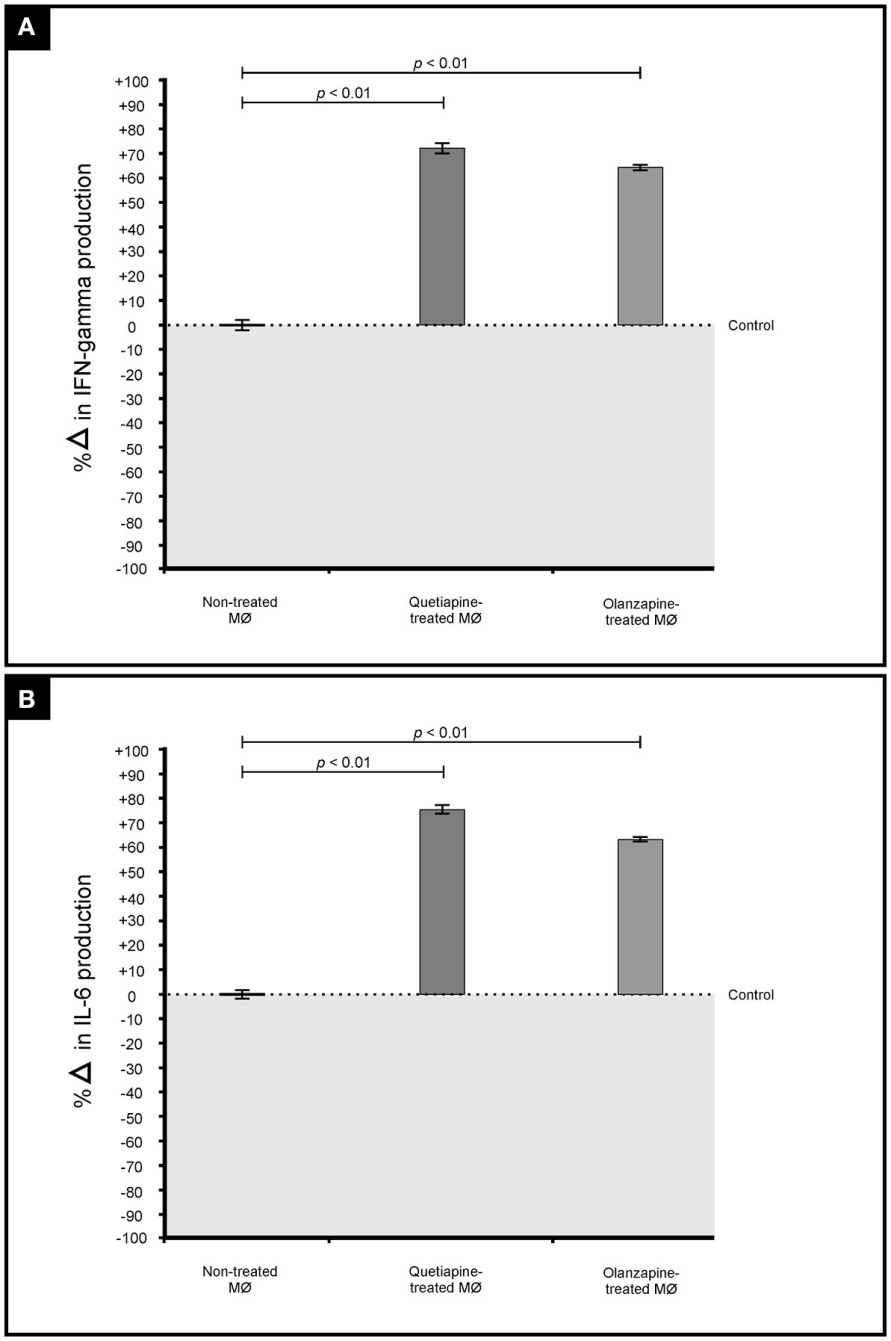
**Effect of quetiapine and olanzapine on the immunological response of macrophages**. Drug treatment induced challenged macrophages to produce significantly (*p* = 0.01) more pro-inflammatory cytokines i.e., interferon gamma **(A)** and interleukin 6 **(B)** when compared to non-treated macrophages.

**Figure 6:**
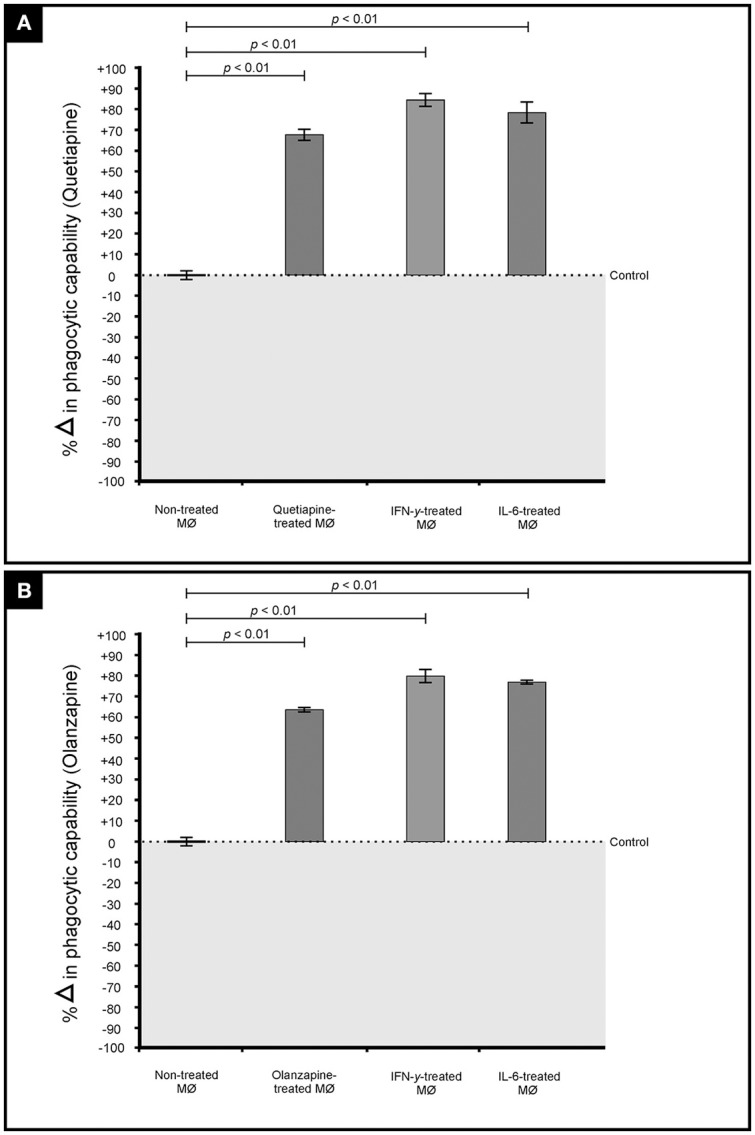
**Effect of quetiapine (A)** and olanzapine **(B)** in chemosensitising macrophage (MØ) to phagocytose cryptococcal cells. Drug treatment sensitized challenged macrophages to significantly (*p* < 0.01) internalize cryptococcal cells when compared to non-treated cells. A similar response i.e., increased internalization, was observed when macrophages were stimulated (challenged) with interferon gamma and interleukin 6.

The primary function of macrophages is to sense and resolve threats such as invading cryptococcal cells through the process of phagocytosis (Voelz and May, 2010). It therefore became important to assess how these drugs may influence the functioning of macrophages. First, the production of pro-inflammatory cytokines by macrophages when challenged with cryptococcal cells, in the presence of quetiapine or olanzapine, was assayed. The obtained results showed that macrophages produced significantly (*p* < 0.05) more IFN-γ (Figure 5A) and IL-6 (Figure 5B) when treated with respective anti-psychotic drugs compared to when they were not treated. These pro-inflammatory cytokines are reported in literature to recruit macrophages and enhance their phagocytic action—functions that are pivotal in the clearance of infecting cells (Bonham et al., 2008). Second, it was sought to determine if the test drugs may also sensitize macrophages to internalized and trap more cryptococcal cells inside phagolysosomes compared to in the absence of either drug. Here, it was determined that both drugs significantly (*p* < 0.05) enhanced the ability of macrophages to internalize more cells (Figure 6). To be specific, quetiapine enhanced internalization by 65% (Figure 6A) while olanzapine enhanced it by 63% (Figure 6B) when compared to the internalization of cryptococcal cells by macrophages in the absence of quetiapine or olanzapine. For reference purposes, the internalization of cryptococcal cells by macrophages was also assayed in the presence of either IFN-γ or IL-6 (Figures 6A,B). As expected, the two cytokines significantly (*p* < 0.05) enhanced internalization of cryptococcal cells when compared to macrophages not treated/stimulated with cytokines (Figure 6). It was, thus, interesting to note that the response of macrophages to the test drugs mirrored the macrophage response to the two cytokines.

## Discussion

The need for alternative anti-fungal drugs, to better manage disseminated cryptococcal infections, necessitated the current study. At the moment, conventional drugs such as fluconazole and amphotericin B are routinely used in South Africa to control cryptococcal infections (Jarvis and Meintjes, 2011; Govender and Dlamini, 2014). However, there are issues associated with these conventional drugs such as the fungistatic nature of fluconazole or undesired effects of amphotericin B (Roemer and Krysan, 2014). To address these issues, it is not surprising to see an increase in the number of articles wherein authors considered repurposing non-traditional antifungals to control fungal growth (Cederlund and Mardh, 1993; Sebolai and Ogundeji, 2015). An important aspect in these articles is that the authors followed standard protocol for assessing *in vitro* susceptibility (i.e., EUCAST or CLSI), which provided insight into the effectiveness of repurposed drugs (Sebolai and Ogundeji, 2015). In one such a study, the authors considered repurposing aspirin, which is traditionally used as an anti-inflammatory drug, as an anti-Cryptococcus drug (Ogundeji et al., 2016). Moreover, the authors used aspirin at concentrations that were within the recommended dosage in the blood to deliver therapeutic benefits (Levy, 1976).

In the current study, anti-psychotic drugs were considered as suitable candidate drugs. When disseminated, cells can scavenge precursors from the brain tissue to form melanized cell walls (Buchanan and Murphy, 1998). The result of which, is a cell that can be protected against host effector mechanisms (Polacheck et al., 1990) and successfully manifest a condition wherein a patient is left with an altered mental state. From the above, it is clear that the localization of infection should be an important consideration as a drug that is able to cross the blood brain barrier may be a viable treatment option in case of a disseminated cryptococcal infection. At the same time, anti-psychotic drugs may have an ancillary benefit of managing pathogen-emergent psychosis.

We have successfully demonstrated that both quetiapine and olanzapine have anti-Cryptococcus activity. It was also significant to note that this activity was displayed at concentrations, for each drug, which were within the recommended dosage in the blood (Skov et al., 2015; Lu et al., 2016). Moreover, both drugs were shown to individually effect synergism when paired with either fluconazole or amphotericin B. Interestingly, in their study, Vitale and co-authors reported that chlorpromazine and trifluopherazine (dopamine antagonist drugs that are prescribed to treat schizophrenia) displayed antifungal activity against some fungal species (Vitale et al., 2007). However, these authors did not elucidate on the possible mode of action employed by these drugs. In the same year, a paper by Maruoka and co-authors suggested it is reasonable that anti-psychotic drugs may target membranes and lead to increased permeability (Maruoka et al., 2007). We were able to arrive to a similar conclusion as Maruoka and co-authors—that is, drug treatment lead to the inability of membranes to control flow of molecules across the membrane leading to cell death. It now becomes pivotal to also demonstrate if these drugs, which targeted eukaryotic membranes, would yield the desired outcome without attacking the host membranes. Toward this end, the effectiveness of these drugs should be determined in laboratory animals.

Another interesting finding was that the drugs chemically sensitized macrophages (much like cytokines) to, in turn, phagocytose more cryptococcal cells. This may be critical to kill disseminating cells that are “parasitic” to or “hiding” inside macrophages, even melanised cells that may be resistant to macrophage-induced oxidative stress. In another study, it was shown that parasitised macrophages can also be sensitized using chloroquine (anti-malarial drug) to kill parasitic cryptococcal cells by forming iron-complexes (Webber et al., 2000). Thus, targeting or sensitizing of macrophage metabolism may be a novel therapeutic strategy against parasitic pathogens with immense clinical applications.

## Author contributions

AO and OS designed the study. AO performed the experiments. OS provided the facilities and resources to conduct the study. AO, OS, and CP wrote the manuscript.

## Funding

This work was supported by a grant from the National Research Foundation of South Africa (UID 87903) and the University of the Free State.

### Conflict of interest statement

The authors declare that the research was conducted in the absence of any commercial or financial relationships that could be construed as a potential conflict of interest.
